# Inactive Gingipains from *P. gingivalis* Selectively Skews T Cells toward a Th17 Phenotype in an IL-6 Dependent Manner

**DOI:** 10.3389/fcimb.2017.00140

**Published:** 2017-04-27

**Authors:** Izabela Glowczyk, Alicia Wong, Barbara Potempa, Olena Babyak, Maciej Lech, Richard J. Lamont, Jan Potempa, Joanna Koziel

**Affiliations:** ^1^Department of Microbiology, Faculty of Biochemistry, Biophysics and Biotechnology, Jagiellonian UniversityKrakow, Poland; ^2^Center for Oral Health and Systemic Disease, University of Louisville School of Dentistry, University of LouisvilleLouisville, KY, USA; ^3^Department of Nephrology, Klinikum der Ludwig-Maximilians-Universität München, Medizinische Klinik und Poliklinik IVMunich, Germany

**Keywords:** *P*. *gingivalis*, gingipains, dendritic cells, Th17, IL-6

## Abstract

Gingipain cysteine proteases are considered key virulence factors of *Porphyromonas gingivalis*. They significantly influence antibacterial and homeostatic functions of macrophages, neutrophils, the complement system, and cytokine networks. Recent data indicate the role of *P. gingivalis* in T cell differentiation; however, the involvement of gingipains in this process remains elusive. Therefore, the aim of this study was to investigate the contribution of danger signals triggered by the gingipains on the generation of Th17 cells, which play a key role in protection against bacterial diseases but may cause chronic inflammation and bone resorption. To this end we compared the effects of the wild-type strain of *P. gingivalis* (W83) with its isogenic mutant devoid of gingipain activity (ΔKΔRAB), and bacterial cells pretreated with a highly-specific inhibitor of gingipains activity (KYTs). Antigen presenting cells (APCs), both professional (dendritic cells), and non-professional (gingival keratinocytes), exposed to viable bacteria expressed high amounts of cytokines (IL-6, IL-21, IL-23). These cytokines are reported to either stimulate or balance the Th17-dependent immune response. Surprisingly, cells infected with *P. gingivalis* devoid of gingipain activity showed increased levels of all tested cytokines compared to bacteria with fully active enzymes. The effect was dependent on both the reduction of cytokine proteolysis and the lack of cross-talk with other bacterial virulence factors, including LPS and fimbriae that induce *de novo* synthesis of cytokines. The profile of lymphocyte T differentiation from naive T cells showed enhanced generation of Th17 in response to bacteria with inactive gingipains. Moreover, we found that gingipain-dependent induction of Th17 cells was highly specific, since other T cell-subsets remained unchanged. Finally, inhibition of IL-6 signaling in dendritic cells led to a significant depletion of the Th17 population. Cumulatively, this study revealed a previously undisclosed role of gingipain activity in the process of Th17 differentiation reliant on blocking signaling through IL-6. Since inactivation of gingipains accelerates the skewing of T cells toward Th17 cells, which are detrimental in periodontitis, IL-6 signaling may serve as an attractive target for treatment of the disease.

## Introduction

Periodontitis is a chronic inflammatory disease affecting tissues surrounding and supporting the teeth, called the periodontium. The disease is initiated by pathogenic bacteria proliferating in a biofilm below the gum line, which stimulate a local inflammatory reaction. Deregulation of the immune system leads to sustained inflammation, tissue destruction, and gradual degradation of the tooth-supporting alveolar bone (Di Benedetto et al., 2013). Among over 500 bacterial species that inhabit the oral cavity, only a handful are associated with periodontitis and can be defined as periodontal pathogens. These include *Porphyromonas gingivalis* (Socransky et al., 1998), which together with *Treponema denticola* and *Tannerella forsythia* form “the red complex” that is strongly implicated in the initiation and progression of chronic periodontitis (Holt and Ebersole, 2005). *P. gingivalis* expresses a variety of virulence factors, including fimbriae, lipopolysaccharide, and cysteine proteases—gingipains. The latter are considered major contributors to the pathogenic potential of *P. gingivalis* (Guo et al., 2010). Moreover, gingipains have been identified in all clinical isolates, and their expression level correlates with exacerbation of the disease. Gingipains strongly influence components of the innate and adaptive immune system (Ismail et al., 2015). For example gingipains contribute to hyporesponsiveness of macrophages during infection, reducing the expression of CD14 molecules and diminishing bacterial recognition (Wilensky et al., 2015). Moreover, gingipains' proteolytic activity-dependent modification of the neutrophil surface leads to impaired clearance of these cells once they become apoptotic (Guzik et al., 2007). Together, such effects on phagocytic cells augment the inflammatory reaction in the periodontium, which is further enhanced by de-regulation of complement system activation and function (Popadiak et al., 2007; Potempa et al., 2009), and modification of activity of some cytokines, such as IL-8, INF-γ, TNF-α, IL-1, CXCL8, and CXCL10 (Yun et al., 2001; Uehara et al., 2008; Moelants et al., 2014). Finally, gingipains also affect the adaptive immune system as exemplified by modulation of T cell function due to hydrolysis of CD4 and CD8 molecules (Kitamura et al., 2002) and efficient cleavage of antibodies (Vincents et al., 2011).

The chronic inflammatory reaction observed in periodontitis patients is supported by the altered activation of T lymphocytes, thus influencing the production of antibodies by B cells. CD4^+^ Th cells are major regulators of the adaptive immune system. They can differentiate into a variety of effector T cell subsets, such as Th1, Th2, and Th17. Their phenotype depends on the presence of stimulatory ligands and the cytokine milieu. A critical role for IL-17 and Th17 cells in some pathologies is illustrated in autoimmune diseases such as psoriasis, psoriatic arthritis, or rheumatoid arthritis (Tesmer et al., 2008). Furthermore, an increasing body of evidence indicates that Th17 lymphocytes can efficiently promote osteoclastogenesis and bone resorption in periodontitis (Gaffen and Hajishengallis, 2008; Okamoto and Takayanagi, 2011; Moutsopoulos et al., 2012). The major cytokine secreted by Th17 cells is IL-17, which influences both immune and non-immune cells. This consequently activates proinflammatory signaling pathways, along with the production of cytokines, chemokines and matrix metalloproteinases (Witowski et al., 2004; Kramer and Gaffen, 2007). Moreover, an interaction of IL-17 and Del-1 plays an important role in the recruitment of neutrophils, that contributes to bone destruction (Eskan et al., 2012). Together these findings expand the classical framework of the Th1/Th2 paradigm in describing the pathogenesis of periodontitis (Gaffen and Hajishengallis, 2008).

The differentiation of human naive CD4^+^ T cells into the population of Th17 cells is regulated by polarizing cytokines, such as IL-1, IL-6, IL-21, and IL-23 (Manel et al., 2008). These cytokines are secreted by antigen-presenting cells in response to pathogen-associated microbial patterns (PAMPs). Among them, IL-6 plays a critical role in the differentiation of Th17 cells with simultaneous inhibition of regulatory T cells (Treg; Kimura and Kishimoto, 2010). Importantly, IL-6 is also one of the major target-cytokines for IL-17 signaling, which amplifies the effects of IL-17 mediated pathology and supports maintenance of Th17 cells by up-regulating expression of IL-23, IL-21, IL-23R, and RANKL (Scheller et al., 2011). In line with this, several studies in humans have shown elevated levels of IL-6 and IL-17 in inflamed gingiva, gingival crevicular fluid and plasma in patients with periodontitis (Loos, 2005). Due to the crucial role of IL-6 in skewing lymphocytes toward Th17 phenotype, blocking of IL-6 function may become an attractive target for treatment of chronic inflammatory disease such as periodontitis. Therefore, taking into account the important role of the gingipains secreted by *P. gingivalis* in the pathogenesis of periodontitis, here we studied their contribution to regulation of the IL-6-Th17 axis.

## Materials and methods

### Cell preparation and cultures

Monocyte-derived dendritic cells (moDCs) were obtained from PBMCs, which were isolated from human peripheral blood by using density gradient centrifugation. Monocytes were purified from the mononuclear cell layer using BD Human Monocyte Enrichment Set—DM (BD IMag). Cells (2 × 10^6^ cells suspended in 2 ml) were cultured in RPMI 1640 medium (Life Technologies) supplemented with 10% FBS (Life Technologies) and 100 U/ml Penicillin-Streptomycin (Life Technologies). moDCs were generated by adding 10 ng/ml GM-CFS and 500 U/ml IL-4. Cultures were fed every 2 days by replacing medium with the fresh supplemented with a full set of cytokines.

Lymphocytes were isolated from the PBMC fraction by using the BD Human Naive CD4+ T Cell Enrichment Set—DM (BD IMag) and cultured in RPMI 1640 medium (Life Technologies) supplemented with 10% FBS (Life Technologies) and 100 U/ml Penicillin-Streptomycin (Life Technologies). Blood was collected at the Regional Blood Center, Krakow, Poland, from healthy donors who provided written informed consent for the collection of samples and subsequent cell isolation and analysis. For human subject confidentiality assurances, blood material was de-identified thus this manuscript adheres to appropriate exclusions from human subject approval. TIGKs (telomerase-immortalized gingival keratinocytes; Moffatt-Jauregui et al., 2013), were routinely cultured in KBM-Gold TM keratinocyte basal medium supplemented with Single Quotes TM (Lonza).

### Bacterial growth

*P. gingivalis* W83, and ΔKΔRAB were grown under anaerobic conditions (90% N_2_, 5%CO_2_, 5%H_2_) at 37°C on blood (5% v/v sheep blood) agar plates or in liquid Scheadler broth (BTL, Lodz, Poland) supplemented with haemin (5 μg/ml; Sigma Aldrich), L-cysteine (50 μg/ml; Sigma Aldrich), menadione (0.5 μg/ml; Sigma Aldrich), and in case of ΔKΔRAB, additionally with tetracycline (1 μg/ml). Bacteria from an overnight culture were centrifuged (4500 g, 10 min), the bacterial pellet was washed three times with phosphate-buffered saline (0.14M NaCl, 0.0026M KCl, 0.01M Na_2_HPO_4_, 0.002M KH_2_PO_4_), pH 7.4 (PBS), and resuspended in PBS. Bacterial cell counts were standardized to an optical density of 1.0 at 600 nm corresponding to 1 × 10^9^ CFU/ml.

### Stimulation of moDCS and keratinocytes with *P. gingivalis*

Dendritic cells and keratinocytes (at a concentration of 0.6 × 10^6^/ml) were left untreated (control) or infected with *P. gingivalis* W83 or ΔKΔRAB at multiplicity of infection MOI 1:25, 1:50, 1:100, 1:200 (human cells/*P. gingivalis*) in the presence or absence of KYT-1 and KYT-36 (each at 1 μM), specific inhibitors of gingipains (Peptide Institute Inc.) (Kadowaki et al., 2004). The inhibitors were added to cultures 15 min before stimulation of cells. Infected moDCs were cultured for 6 and 24 h, and then used for experiments with T lymphocytes. Both moDCs and TIGKs infected with *P. gingivalis* were cultured for 2, 6, and 24 h, followed by harvesting for RNA isolation, or collection of culture medium for assay of cytokine levels.

### Gingipain activity measurement

After 2, 6, and 24h of stimulation of moDC with *P. gingivalis*, a sample of conditioned medium was withdrawn, centrifuged (12000 × g; 5 min) and 10 μl of supernatant was added to 90 μl TNCT buffer (50 mM Tris pH 7.5, 150 mM NaCl, 5 mM CaCl_2_, 0.05% Tween-20) with 20 mM L-cysteine followed by 100 μl of 0.2 mM substrate solution. Arginine- and lysine-specific and gingipain activities were determined with L-BA*p*Na and N-(*p*-Tosyl)-Gly-Pro-Lys-4-nitroanilide as substrates, respectively. Substrate hydrolysis was recorded at 405 nm for 40 min and activity expressed as mOD/min/1 μl of supernatant.

### Activation and inactivation of gingipain

HRgpA was activated in TNC buffer (50 mM Tris, 150 mM NaCl, 5 mM CaCl_2_, pH 7.5) supplemented with 20 mM L-cysteine for 15 min in 37°C. To test the effect of inactivated gingipains, enzyme samples were treated with specific gingipain inhibitors KYT-1 and KYT-36 (each at 1 μM) for 15 min in 37°C before stimulation of the cells. Cultured media for the cells stimulated with gingipains contained 10 mM L-cysteine.

### Stimulation of moDCS with virulence factors from *P. gingivalis*

Dendritic cells at a concentration of 0.6 × 10^6^/ml were left untreated (control) or stimulated with Ultra Pure LPS from *P. gingivalis* (InvivoGen) [2.5 μg/ml], FimA purified from ATCC 33277 [10 μg/ml] or both together. Additionally, active or inhibitor-treated HRgpA [2 nM] was mixed with LPS or FimA and applied to moDC. Cells were stimulated for 4 h before culture medium was collected for assay of IL-6 levels and the cells harvested for RNA isolation.

### Quantitative reverse transcription PCR

Total cellular RNA was extracted from moDCs, TIGK, and T cells using TRIzol Reagent, according to the manufacturer's instructions. cDNA was prepared by reverse transcription (RT) using the High-Capacity cDNA Reverse Transcription Kit (Applied Biosystems). Seven hundred nanograms of RNA from each sample was used for cDNA synthesis with oligo (dT) primers, according to the manufacturer's instructions. The quantitative PCR (qPCR) reaction was performed with a SYBR Green method in a reaction volume of 20 μl, containing 0.3 μl (moDC, T cells) or 1 μl (TIGKs) of cDNA sample, 10 μM of each primer and 1 × SYBR Green JumpStart Taq Ready Mix. qRT-PCR was performed by using forward and reverse primers for transcripts of listed genes (Table 1), and for the housekeeping EF2 gene (used for normalization). After 5 min of initial denaturation at 95°C, reactions were carried out for 40 cycles, followed by a final elongation step at 72°C for 10 min. Primer sequences and conditions for denaturation (1), annealing (2), and extension (3) for each pair of primers are provided in Table 1. Means for threshold cycle (C_*t*_) values were calculated and analyzed using the “Δ-ΔC_*t*_” quantification method (Livak and Schmittgen, 2001).

**Table 1:** **Oligonucleotide sequences used in the quantitative reverse transcription polymerase chain reaction (qRT-PCR)**.

**Oligonucleotide**	**Sequence**	**Program**
*EF2 F*	5′-GACATCACCAAGGGTGTGCAG-3′	(1) 95°C, 30s (2) 56°C, 30s (3) 72°C, 45s
*EF2 R*	5′-TTCAGCACACTGGCATAGAGGC-3′	
*IL-6 F*	5′-AAATTCGGTACATCCTCGACGGCA-3′	
*IL-6 R*	5′-AGTGCCTCTTTGCTGCTTTCACAC-3′	
*IL-21 F*	5′-GACTTGGTCCCTGAATTTCTGC-3′	
*IL-21 R*	5′-CCTGCATTTGTGGAAGGTGG-3′	
*IL-23 F*	5′-GCTTTCACAGAAGCTCTGCAC-3′	
*IL-23 R*	5′-AGACCCTGGTGGATCCTTTG-3′	
*IL-1β F*	5′-GATGTCTGGTCCATATGAACTG-3′	
*IL-1β R*	5′-TTGGGATCTACACTCTCCAGC-3′	
*IL-12p35 F*	5′-ATGATGGCCCTGTGCCTTAG-3′	
*IL-12p35 R*	5′-TCCGGTTCTTCAAGGGAGGA-3′	
*IL-12p40 F*	5′-ATTCTGCGTTCAGGTCCAGG-3′	
*IL-12p40 R*	5′-AGAACCTAACTGCAGGGCAC-3′	
*TNF F*	5′-GTCAGATCATCTTCTCGAACCCCGA-3′	
*TNF R*	5′-CAGGGCAATGATCCCAAAGTAGA-3′	
*IL-6R F*	5′-AGCCTCCCAGTGCAAGATTC-3′	
*IL-6R R*	5′-GCATGCTTGTCTTGCCTTCC-3′	
*IL-1R F*	5′-AGGTAGACGCACCCTCTGAA-3′	
*IL-1R R*	5′-GCATTTATCAGCCTCCAGAGAAG-3′	
*TGFβR F*	5′-AGCGGTCTTGCCCATCTTC-3′	
*TGFβR R*	5′-GGGGCCATGTACCTTTTTGTT-3′	
*RORC F*	5′-GACAGGGCCCCACAGAGA-3′	
*RORC R*	5′-GAAGAAGCCCTTGCACCCC-3′	
*RORα F*	5′-AGCCAGGCAGCAGCG-3′	
*RORα R*	5′-AAAAAGCCCTTGCAGCCTCC-3′	
*STAT3 F*	5′-GAAACAGTTGGGACCCCTGA-3′	
*STAT3 R*	5′-AAGCGGCTATACTGCTGGTC-3′	
*RUNX1 F*	5′-GGTTTCGCAGCGTGGTAAAA-3′	
*RUNX1 R*	5′-TGGCATCGTGGACGTCTCTA-3′	
*IRF4 F*	5′-CCATGACAACGCCTTACCCT-3′	
*IRF4 R*	5′-CCTGTCACCTGGCAACCATT-3′	
*BATF F*	5′-CCCTGGCAAACAGGACTCAT-3′	
*BATF R*	5′-GATCTCCTTGCGTAGAGCCG-3′	
*IFNγ F*	5′-GGCTTTTCAGCTCTGCATCG-3′	(1) 95°C, 30s (2) 60°C, 60s (3) 72°C, 45s
*IFNγ R*	5′-TTCTGTCACTCTCCTCTTTCCAA-3′	
*IL-8 F*	5′ -ATGACTTCCAAGCTGGCCGTGGCT-3′	
*IL-8 R*	5′ -TCTCAGCCCTCTTCAAAAACTTCT-3′	

### Cytokine assay

The level of IL-6, IFNγ, TNF (BD Bioscience), and IL-23 (Novex Invitrogen) was determined by using a commercially available ELISA kit, according to the manufacturer's instructions. IL-1β, IL-12p70, IL-8, and IL-10 was determined by using the CBA Human Inflammatory Cytokine Kit (BD Bioscience) according to the manufacturer's instructions and results were analyzed by FCAP Array v2.0.2.

### Proliferation of T cells

For a proliferation assay, lymphocytes were stained with CSFE according to the CellTrace™ CFSE Cell Proliferation Kit Protocol (Molecular Probes). Lymphocytes (at a concentration of 1.7 × 10^5^/ml) were added to the moDCs cells (0.5 × 10^5^/ml), which were previously non-infected or infected with *P. gingivalis*. Proliferation was evaluated on day 1, 3, 5, and 7 after co-culturing with moDCs. Data were acquired by the FACSCalibur system (Becton Dickinson) and analyzed by FlowJo V10 software.

### Differentiation of Th cells

After 3, 5, or 7 days co-culture of moDCs with lymphocytes, T cells were stimulated with PMA (50 ng/ml) and ionomycin (500 ng/ml) in the presence of Golgi Stop (BD Biosciences), and collected for staining. For intracellular staining, a cytofix/cytoperm kit was used (Becton Dickinson) with fluorescently labeled antibodies against transcription factors: T-bet—Alexa Fluor®488, GATA3- Alexa Fluor®488, ROR-γt-PE, Fox-P3-Alexa Fluor®488, and cytokines: INF-γ-Alexa Fluor® 647, IL-4-APC, IL-17A-PerCP-Cy5.5. Data were acquired by the FACSCalibur system (Becton Dickinson) and analyzed by FlowJo V10 software.

### Evaluation of Th17 signaling pathway molecules gene expression

Dendritic cells (0.6 × 10^6^/ml) were stimulated for 6 h with *P. gingivalis* strains at MOI 1:50. Lymphocytes (2 × 10^6^/ml) were added, and after 1, 2, and 3 days of co-culture they were gently collected, centrifuged (280 × g, 10 min), and lysed with TRIzol Reagent. RNA was isolated and the relative expression of genes *IL-6R, IL-1R, TGF*β, *RORc, ROR*α, *STAT3, RUNX1, IRF4, BAFT* to the reference house-keeping gene *EF2* was measured by using the qRT-PCR method described above.

### Evaluation of STAT3 and IRF4 activation

Three, 4 and 5 days after co-culture of moDCs with lymphocytes, T cells were stimulated with PMA (50 ng/ml) and ionomycin (500 ng/ml) in the presence of Golgi Stop (BD Biosciences), and collected for staining. For intracellular staining, a cytofix/cytoperm kit was used (Becton Dickinson) with fluorescently labeled antibodies against transcription factors: Alexa Fluor®488 Mouse Anti-Stat3 (pY705; BD Biosciences) and PE-IRF4 Monoclonal Antibody (3E4)(eBioscience). Data were acquired by the FACSCalibur system (Becton Dickinson) and analyzed by FlowJo V10 software.

### Statistical analysis

All experiments were performed at least in triplicate, and results were analyzed for statistical significance using Student's *t*-tests or ANOVA. All values are expressed as means ± *SD*, and differences were considered significant at *p* < 0.05.

## Results

### IL-6 expression induced by *P. gingivalis* depends on activity status of gingipains

**Figure 1:**
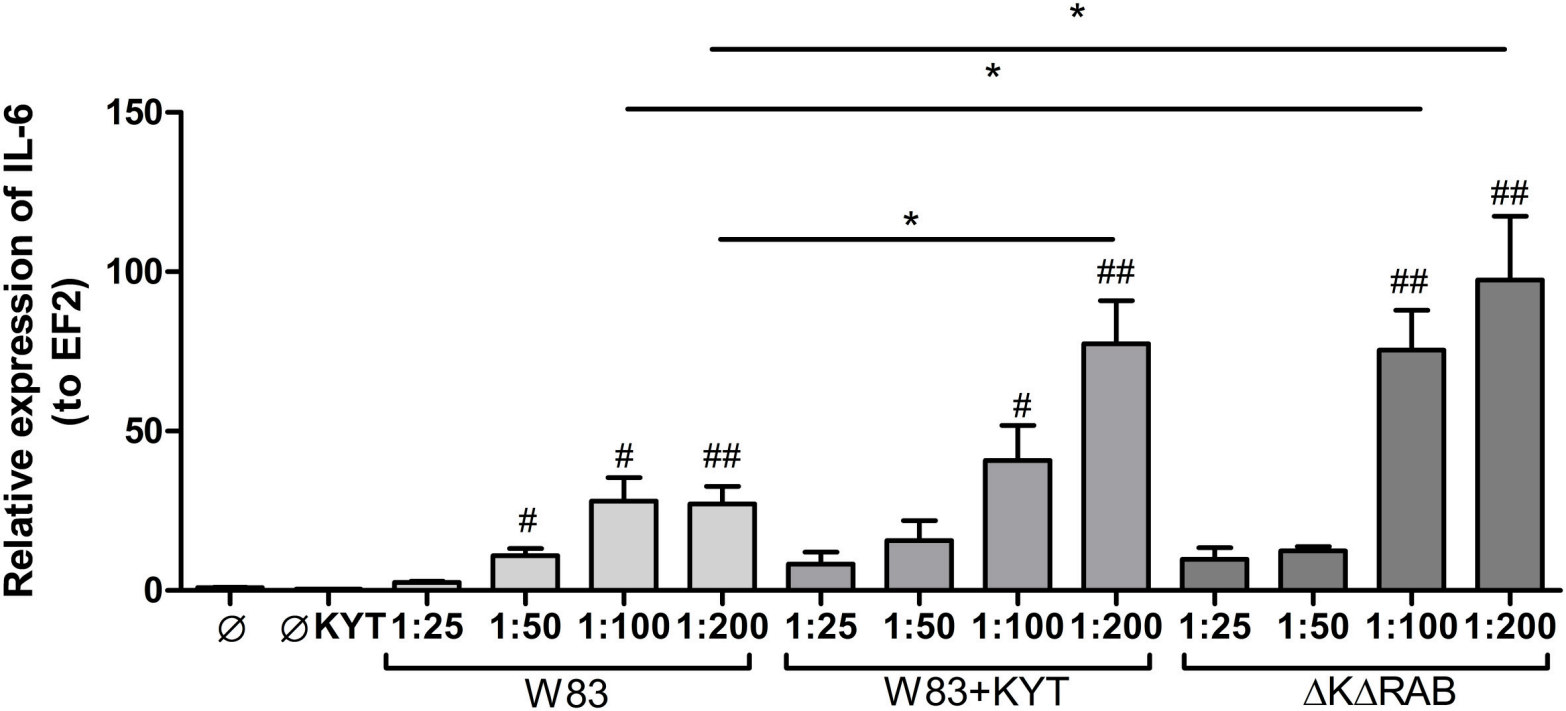
**Expression of interleukin 6 in human monocyte-derived dendritic cells in response to *Porphyromonas gingivalis* infection**. Monocyte-derived dendritic cells (moDC) were untreated or exposed to *P. gingivalis* W83 in the presence or absence of specific protease inhibitors (KYT-1 and KYT-36, each at a concentration of 1 μM) or the isogenic gingipain-null mutant ΔKΔRAB for 2 h. After stimulation, cells were lysed with TRIzol, RNA was isolated and reverse transcriptase PCR was performed. Relative expression of IL-6 was measured by using the Real-Time PCR method. A representative qRT-PCR from three separate experiments performed on moDCs derived from different donors is shown. Data are presented as means ± standard deviations of triplicate assays, and were analyzed with the one-way ANOVA with the Bonferroni post-test correction (#*P* < 0.05, ##*P* < 0.01 vs. control, ^*^P < 0.05 to *P. gingivalis* treated cells).

**Figure 2:**
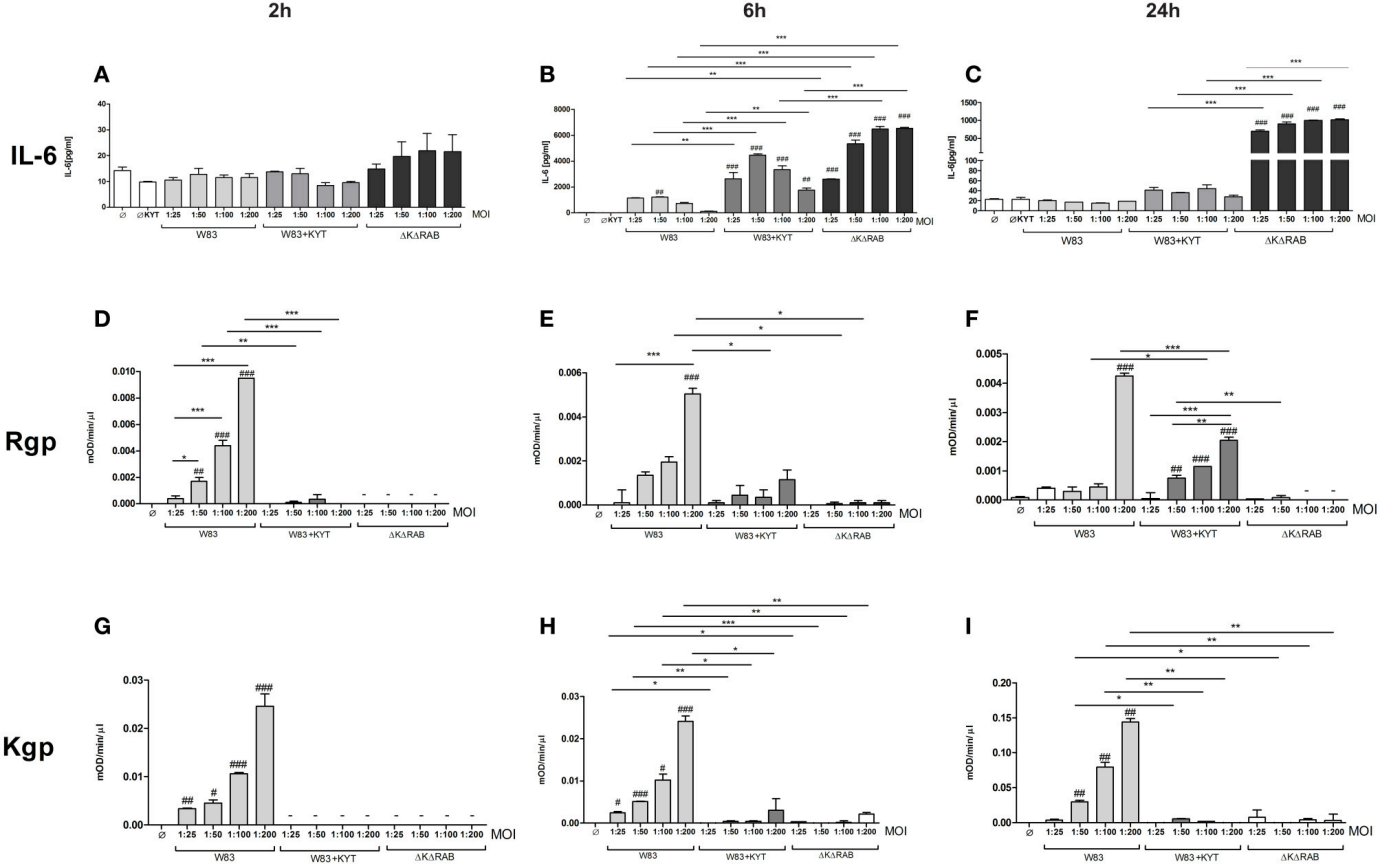
**Interleukin 6 production induced by *P. gingivalis* is inversely dependent on gingipain activity**. Monocyte-derived dendritic cells (moDC) were untreated or exposed to *P. gingivalis* W83 in the presence or absence of specific protease inhibitors (KYT-1 and KYT-36, each at 1 μM concentration) or isogenic *P. gingivalis* gingipain-null mutant ΔKΔRAB for 2 h **(A,D,G)**, 6 h **(B,E,H)**, and 24 h (**C**,**F**,**I**). **(A–C)** Supernatants were collected and the concentration of IL-6 was determined by ELISA. **(D–F)** Arginine-specific and **(G–I)** Lysine-specific gingipain activity was determined using L-BA*p*Na and N-(*p*-Tosyl)-Gly-Pro-Lys-4-nitroanilide acetate salt (200 μM) as a substrate, respectively. Data are presented as means ± standard deviations of assays performed in triplicate using three independent donors, and were analyzed by one-way ANOVA with the Bonferroni post-test correction (^#^*P* < 0.05, ^##^*P* < 0.01, ^###^*P* < 0.001 vs. control, ^*^P < 0.05, ^**^P < 0.01, ^***^P < 0.001 to *P. gingivalis* treated cells).

**Figure 3:**
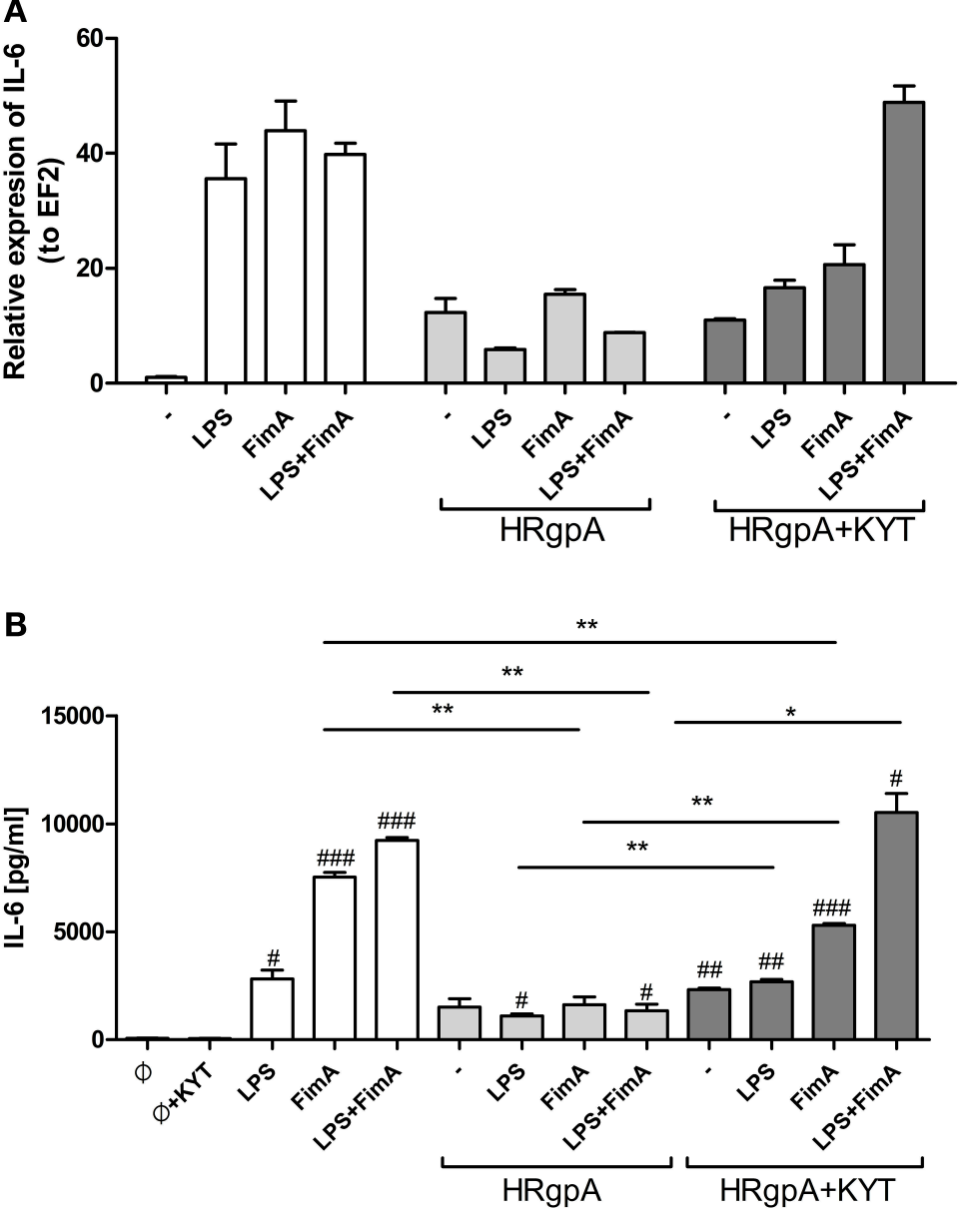
**The role of gingipains in the regulation of cell responses to LPS and fimbriae**. Monocyte-derived dendritic cells (moDC) were untreated or exposed to *P. gingivalis* LPS (2.5 μg/ml), FimA (10 μg/ml) or mixed LPS with FimA. Additionally, cells were co-stimulated with HRgpA (2 nM) in the presence or absence of specific protease inhibitors (KYT-1 and KYT-36, each at a concentration of 1 μM). Four hours after stimulation, culture media were collected and cells were lysed with TRIzol. RNA was isolated and reverse transcriptase PCR was performed. Relative expression of the cytokine gene *IL-6* to the reference house-keeping gene *EF2* was measured by Real-Time PCR. Data show representative fold increase in expression compared to control levels, which were arbitrarily set at 1 **(A)**. Concentration of IL-6 in collected medium was evaluated by standard ELISA method **(B)**; Data are presented as mean ± standard deviation of duplicates from three independent assays and were analyzed with a Student's *t*-test (#*P* < 0.05, ##*P* < 0.01, ###*P* < 0.001 vs. control, ^*^P < 0.05, ^**^P < 0.01 to *P. gingivalis* treated cells).

In response to danger signals expressed by a pathogen, antigen presenting cells, including dendritic cells (DCs), produce inflammatory cytokines. Among them, IL-6 plays a critical role in Th17 differentiation and IL-17 signaling. Since an elevated level of IL-6 was previously reported in inflamed gingiva (Imamura, 2003), we decided to investigate the extent to which DCs-specific cytokine pattern depends on expression and/or activity of gingipains. For that purpose, we used an *in vitro* model of primary human dendritic cells differentiated from blood monocytes (moDCs) as described in the Section Materials and Methods. Briefly, cells were infected with wild-type (WT) *P. gingivalis* W83 expressing gingipains (HRgpA, RgpB, and Kgp) in the presence or absence of KYTs, specific inhibitors of gingipains (Kadowaki et al., 2004), at a multiplicity of infection (MOI) of 1:25, 1:50, 1:100, and 1:200. In parallel, cells were infected with the gingipain-null isogenic mutant (ΔKΔRAB). The expression of IL-6 transcript was measured 2 h post infection (p.i.), whereas the level of secreted IL-6 in conditioned culture media was measured at 2, 6, and 24 h p.i. In this experimental setup *P. gingivalis* was a potent and rapid inducer of IL-6 mRNA expression (2 h) in a MOI-dependent manner for all tested strains and conditions. Surprisingly, the most potent inducers were the gingipain-null strain (ΔKΔRAB) and bacteria with ablated gingipain activity (W83/KYT; Figure 1). The level of secreted IL-6 only weakly corresponded to changes in IL-6 expression observed at the mRNA levels (Figure 2). *P. gingivalis* was a potent inducer of IL-6 secretion in a MOI- and time-dependent manner (Figures 2A–C) but only if gingipain activity was entirely absent (ΔKΔRAB, Figures 2D–I). In cultures infected with WT *P. gingivalis* at a MOI range from 1:25 to 1:200 we found no accumulation of IL-6 in the conditioned medium (Figures 2A–C) apparently due to gingipain activity, which degrades the cytokine (Figures 2D–I). This contention was confirmed by observation of the transient increase of the IL-6 level at 6 h p.i. in cells infected in the presence of KYTs (Figure 2B). At 24 h p.i., however, together with the increase of gingipains' activity (Figures 2D–I), the concentration of IL-6 in the medium dropped to the level detected in non-infected control cells (Figures 2A–C). Taken together, accumulation of IL-6 in conditioned medium of cells infected with *P. gingivalis* was inversely dependent on the presence of active gingipains. Consistent with this, deletion of all three genes encoding gingipains notably up-regulated the IL-6 transcript, when compared to the parental strain with fully active enzymes. To further analyze the mechanism of our observation we estimated the role of both active and inactive enzymes on moDCs activation induced by other *P. gingivalis* virulence factors, such as: LPS and fimbriae. The analysis of IL-6 expression at mRNA and protein levels revealed that synthesis of the cytokine upon stimulation with each cell wall component was strongly inhibited by active gingipains. This indicates that active gingipains interfere with an upstream pathway of stimulation of IL-6 expression (Figures 3A,B). The effect was abrogated when inactive enzymes were used. Cumulatively, the data not only confirm proteolytic degradation of the cytokine (Banbula et al., 1999), but also supports a contribution of gingipains to modulation of immune responses to otherwise stimulatory *P. gingivalis* antigens. Moreover, the results indicate that IL-6 production strongly depends on both gingipain expression and activity.

### The profile of cytokine expression that promotes Th17 differentiation depends on gingipains

**Figure 4:**
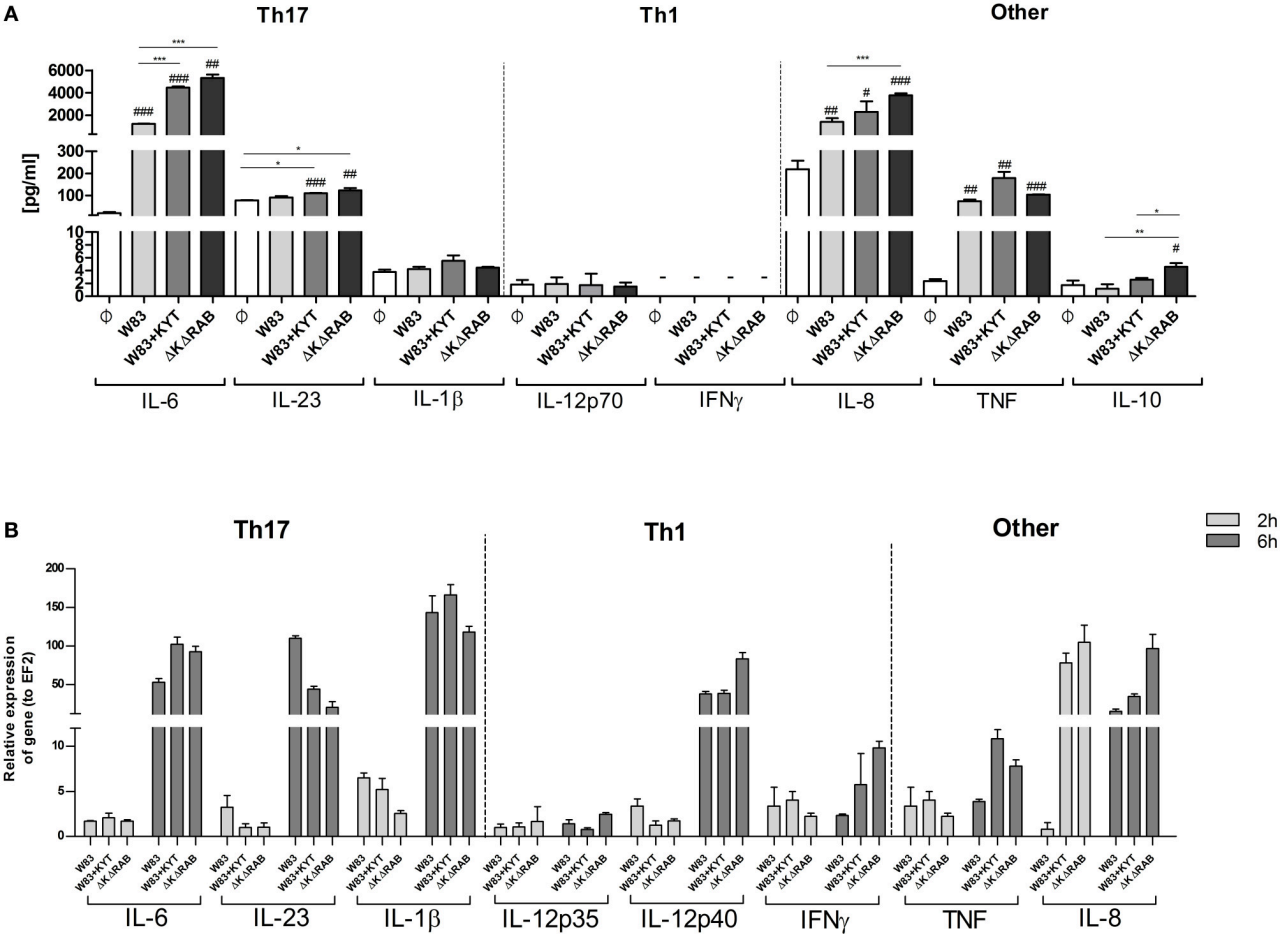
**Gingipain activity differentially determines the mRNA expression and final secretion of cytokines. (A)** Monocyte-derived dendritic cells (moDC) were untreated or exposed to *P. gingivalis* W83 in the presence or absence of specific protease inhibitors (KYT-1 and KYT-36, each at a concentration of 1 μM) or the isogenic gingipain-null mutant ΔKΔRAB at MOI (multiplicity of infection) 1:50. **(A)** Concentrations of cytokines in collected medium were evaluated by ELISA (IL-6, IL-23, TNF, IFNγ) or by using BD CBA Human Inflammatory Kit (IL-1β, IL-12p70, IL-8, IL-10). A representative result from three separate experiments performed on moDCs derived from different donors is shown. Data are presented as mean ± standard deviation of duplicates from three independent assays and were analyzed with a Student's *t*-test (#*P* < 0.05, ##*P* < 0.01, ###*P* < 0.001 vs. control, ^*^P < 0.05, ^**^P < 0.01, ^***^P < 0.001 to *P. gingivalis* treated cells). **(B)** The expression of mRNA for cytokines was evaluated 2 and 6 h post *P. gingivalis* infection by RT-PCR. Data are representative result of fold increase in expression compared to control levels, which were arbitrarily set at 1.

It is suggested that the high level of IL-6 documented in the inflamed periodontal tissue contributes to deregulation of the immune system, which then promotes tissue destruction and disease progression. The detrimental effect of IL-6 can be partly explained by the induction of naive T cell differentiation toward the Th17 phenotype. Since we found that IL-6 upregulation is inversely dependent on activity of gingipains, we decided to estimate the level of other cytokines (IL-23 and IL-1β) known for their regulatory effect on the differentiation of Th17 cells. Infection of moDCs with *P. gingivalis* stimulated secretion of IL-23 and increased accumulation of this cytokine in the conditioned medium but only in the presence of bacteria devoid of gingipain activity (Figure 4A). In the case of IL-1β we observed a similar trend, however the amount of the protein was close to the limit of detection (Figure 4A). Since *P. gingivalis* was described as a pathogen that promotes skewing of T cells into Th1 phenotype (Stashenko et al., 2007; Zeituni et al., 2009), we estimated the level of cytokines critical for that process. The analysis of IL-12 and IFNγ revealed no accumulation of either cytokine in conditioned media (Figure 4A). Further, extended analysis of other pro- and anti- inflammatory cytokines showed upregulation of IL-10, IL-8, and TNFα in moDCs infected with *P. gingivalis*. Interestingly, the levels of IL-8 and IL-10 were inversely dependent on activity of gingipains as we observed for IL-6. Transcriptome analysis revealed an increase in mRNA of all tested cytokines, however only in case of IL-6, INFγ, IL-8, and TNFα was the expression reversely correlated with the activity of gingipains (Figure 4B). The expression of IL-6 was also verified in non-professional antigen presenting cells, human immortalized gingival keratinocytes (TIGKs). Infection of these cells with viable bacteria induced the strong upregulation of the IL-6 transcript (Supplementary Figure 1) that inversely correlated with gingipain activity. Of note, we observed no influence of KYT inhibitors at the applied concentration on cell morphology, viability, proliferation, differentiation, and cytokine expression (data not shown). Taken together, these data indicate that inhibition of gingipains during periodontitis may strongly enhance the secretion of a set of cytokines required for Th17 differentiation in tissues infected with *P. gingivalis*.

### The differentiation of T cells into Th17 phenotype is regulated by gingipains

**Figure 5:**
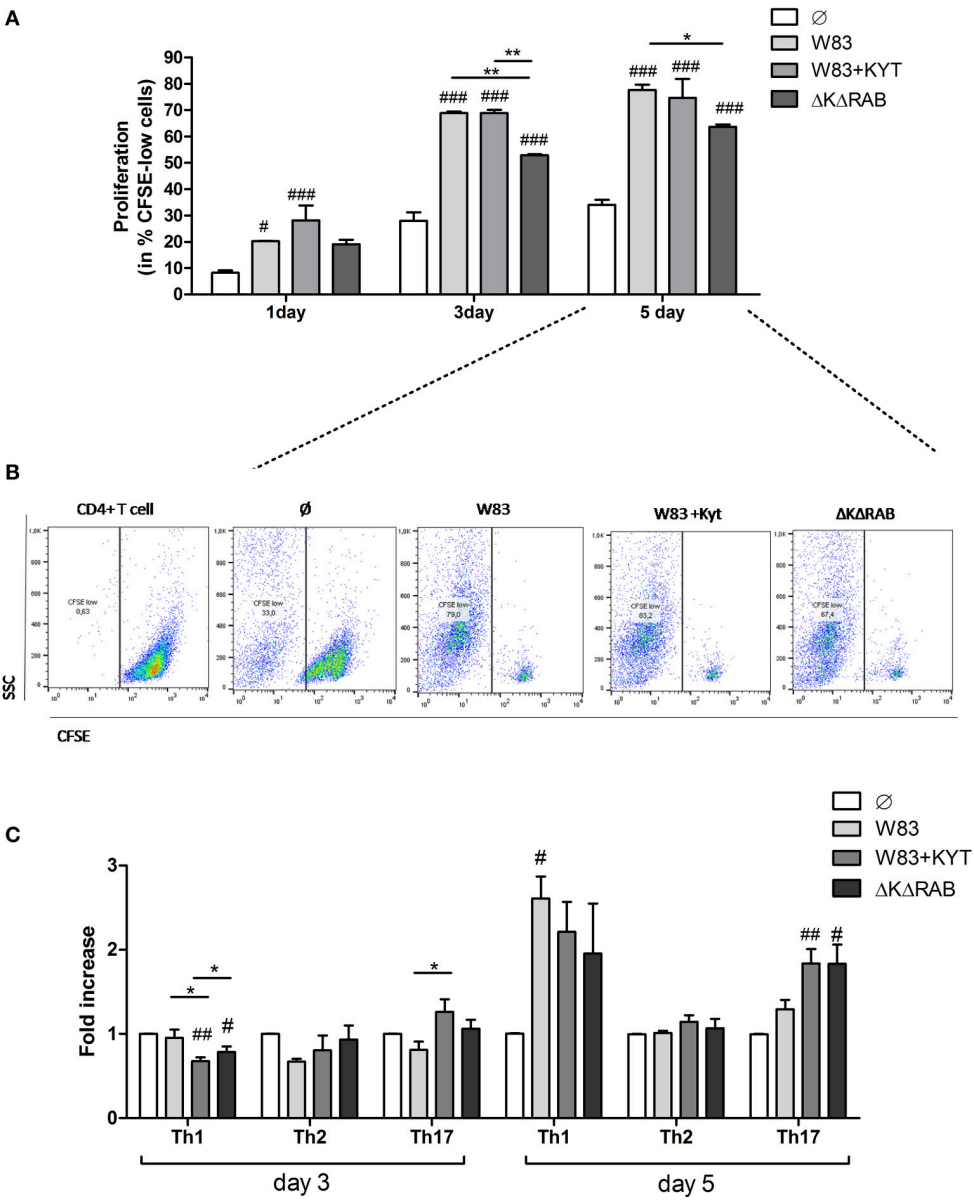
**Proliferation and differentiation of CD4^+^ T cells mediated by gingipains from *P. gingivalis***. Monocyte-derived dendritic cells (moDC) were untreated or exposed to *P. gingivalis* W83 in the presence/absence of specific protease inhibitors (KYT-1 and KYT-36, each at a concentration of 1 μM) or the isogenic gingipain-null mutant ΔKΔRAB for 6 h at MOI (multiplicity of infection) 1:50. Naive CD4^+^ T cells stained with CFSE were added and co-stimulated for 5 days. Proliferation of lymphocytes was measured at day 1, 3, and 5. **(A)** Completed results of proliferation on day 1, 3, and 5 as a percentage of CFSE low cells. Data are presented as a mean ± standard deviation of assays performed in triplicate using four independent donors, and were analyzed with a two way ANOVA with the Bonferoni's posttest correction (#*P* < 0.05, ###*P* < 0.001 vs. control, ^**^P < 0.01 to *P. gingivalis* treated cells) **(B)** Gating strategy for evaluation of T cell proliferation by CFSE dilution. All CD4+ cells having a lower CFSE intensity than intact control lymphocytes were treated as proliferating cells. Data represent the proliferation of lymphocytes on day 5 and show percentage of CFSE low cells. A representative dot plot out of three separate experiments performed on lymphocytes derived from different donors is shown. **(C)** After stimulation with bacteria, the naive CD4^+^ cells were added and co-cultured for an additional 3 or 5 days. At the end of each period, T cells were stimulated with PMA and ionomycin for 4 h in the presence of GolgiStop. For the evaluation of the Th population, cells were stained for intracellular IFN-γ, T-bet (Th1), IL-4, GATA-3 (Th2), IL-17A, RORγt (Th17), and analyzed by flow cytometry. Data show fold increase of the transcription factor and the cytokine positive population, relative to the appropriate control set at 1. Data are presented as a mean ± standard deviation of assays performed in triplicate using cells from three different donors, and were analyzed with a Student's *t*-test (#*P* < 0.05, ##*P* < 0.01 vs. control, ###*P* < 0.001, ^*^P < 0.05 *to P. gingivalis* treated cells).

Based on the finding that gingipains play an important role in the regulation of cytokine expression that promotes induction and/or maintenance of Th17 population, we investigated the potential of these enzymes to influence the proliferation and differentiation of other CD4+ T helper effector cells. To this end, we determined the potential of gingipains to drive T cell proliferation and shift into other Th phenotypes. We infected moDC with WT *P. gingivalis* in the absence and the presence of KYTs to inactivate gingipains. Cells were also infected with the gingipain-null isogenic mutant (ΔKΔRAB). Naive CD4+ T cells were labeled with CSFE and cultured with moDCs 6 h post *P. gingivalis* infection. The proliferation of CD4+ T cells was significantly induced after 1 day of lymphocyte co-culture with moDCs, when compared to the control population of CD4+ T cells, and an increased division rate of cells was sustained up to day 5 (Figures 5A,B). There was no difference in proliferation between cells exposed to moDCs infected with WT *P. gingivalis* or the inhibitor-treated bacteria, whereas the rate of CD4+ T cells proliferation exposed to moDCs infected with the gingipain-null isogenic mutant was significantly reduced, when compared to the WT strain (Figure 5A).

Next, we analyzed co-culture of T cells with *P. gingivalis* infected moDCs for CD4+ T cell differentiation at day 3 and 5. At day 3 only slight changes in the profile of the CD4+ T cell phenotype were observed when compared to control cells (Figure 5C). However, at day 5 a potent induction of Th1 and Th17 populations of CD4+ T helper cells was observed in contrast to the Th2 population, which remained unaffected (Figure 5C). Notably, the highest polarization toward the Th17 phenotype in the population of CD4+ T cells co-cultured with moDCs occurred when the cells were infected with *P. gingivalis* devoid of gingipain activity (Figure 5C).

**Figure 6:**
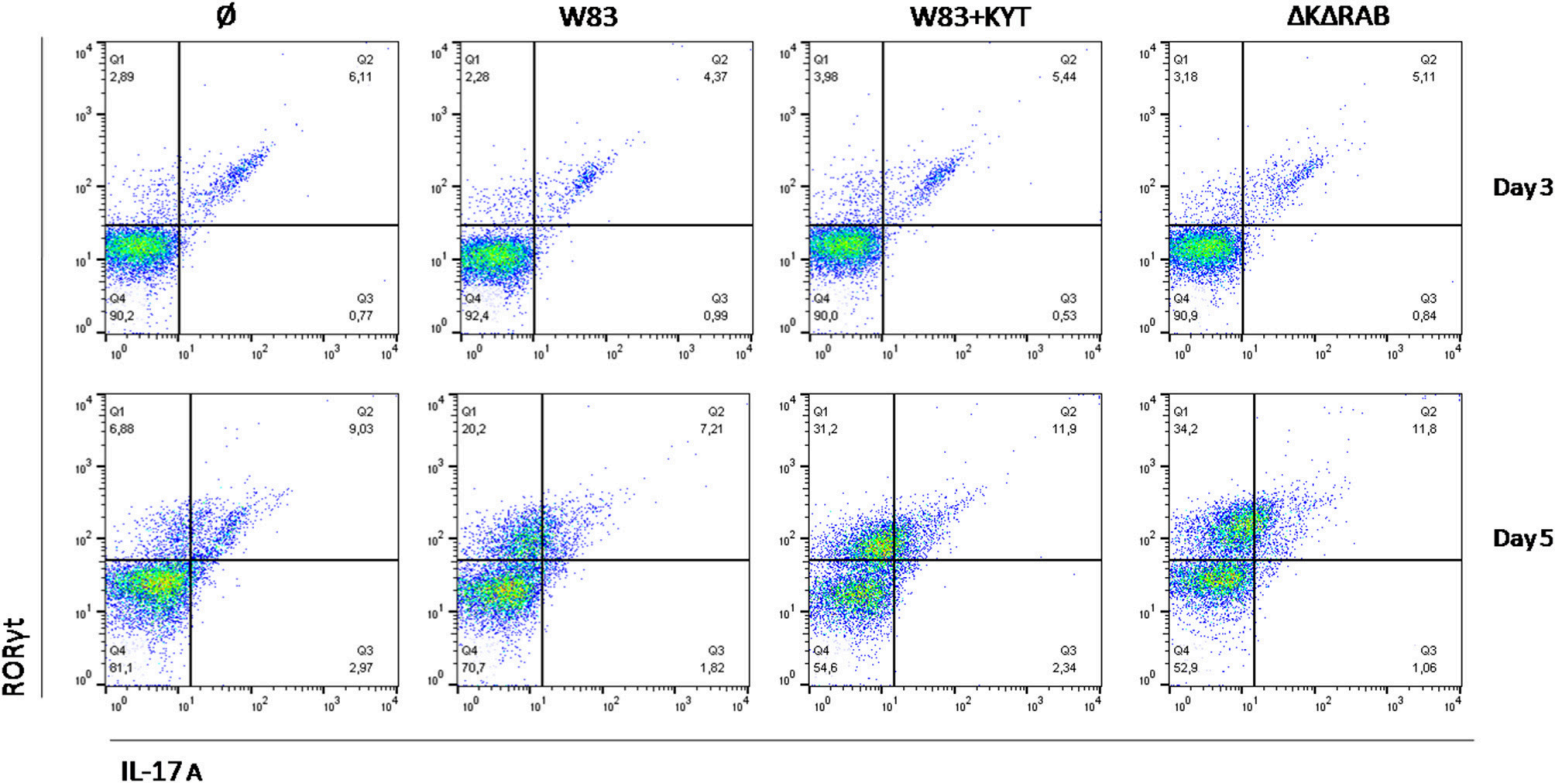
**Th17 cells differentiation depends on gingipain activity**. Monocyte-derived dendritic cells were untreated or exposed to *P. gingivalis* W83 in the presence/absence of specific protease inhibitors (KYT-1 and KYT-36, each at the concentration of 1 μM) for 6 h. Naive CD4^+^ cells were added and co-cultured for an additional 3 or 5 days. At the end of each period, T cells were stimulated with PMA and ionomycin for 4 h in the presence of GolgiStop. For evaluation of the Th17 population, cells were stain for intracellular IL-17A andRORγt, and were analyzed by flow cytometry. Data show a representative dot blot selected from three separate experiments presenting percent of RORγt^+^, IL-17+, RORγt^+^ IL-17^+^ after 3 and 5 days of co-stimulation of naive CD4+ T cells with moDC pulsed with *P. gingivalis*.

Detailed analysis of the Th17 differentiation process revealed robust induction of the Th17 specific transcription factor RORγt (Figure 6), which was observed by day 3 following co-culture. Interestingly, this effect was detected only with bacteria deficient in gingipain activity. The further increase of RORγt positive cells was observed 2 days later (day 5) reaching 2- and 3-fold higher induction levels (over the base line) in the case of infection with the WT strain or with bacteria deficient in gingipains, respectively (Figure 6). The expression of IL-17 was detected at day 5 post co-culture of T-cells with infected moDCs. The highest level of IL-17 positive cells was observed for infections with bacteria that were devoid of gingipain activity (Figure 6), thus corresponding to the activation of the transcription factor.

**Figure 7:**
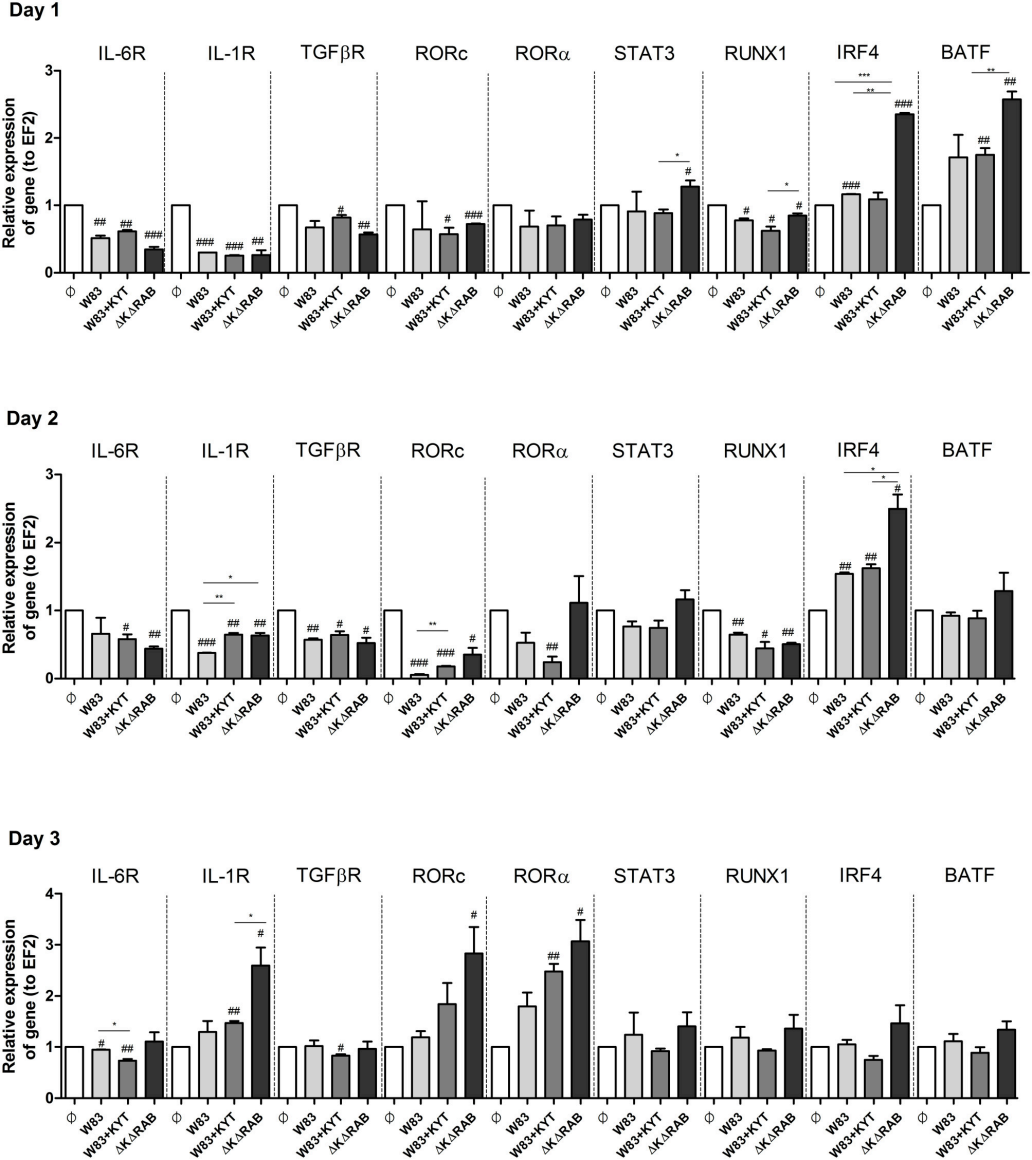
**Activation of Th17 signaling pathway in CD4+ naïve lymphocyte depends on gingipain activity**. Monocyte-derived dendritic cells (moDC) were untreated or exposed to *P. gingivalis* in the presence or absence of specific protease inhibitors (KYT-1 and KYT-36, each at a concentration of 1 μM). After 6 h, CD4+ naïve lymphocytes were added and co-stimulated for 3 consecutive days. At day 1, 2, and 3 after co-incubation, cells were collected and lysed with TRIzol, RNA was isolated and reverse transcriptase PCR was performed. Relative expression of cytokine genes *IL-6R, IL-1R, TGF*β*R, RORc, ROR*α*, STAT3, RUNX1, IRF4, BATF* to the reference house-keeping gene *EF2* was measured by Real-Time PCR. Data represent fold increase in expression compared to control levels, which were arbitrarily set at 1 and were analyzed with a Student's *t*-test (^#^*P* < 0.05, ^##^*P* < 0.01, ^###^*P* < 0.001 vs. control, ^*^P < 0.05, ^**^P < 0.01, ^***^P < 0.001 to *P. gingivalis* treated cells).

**Figure 8:**
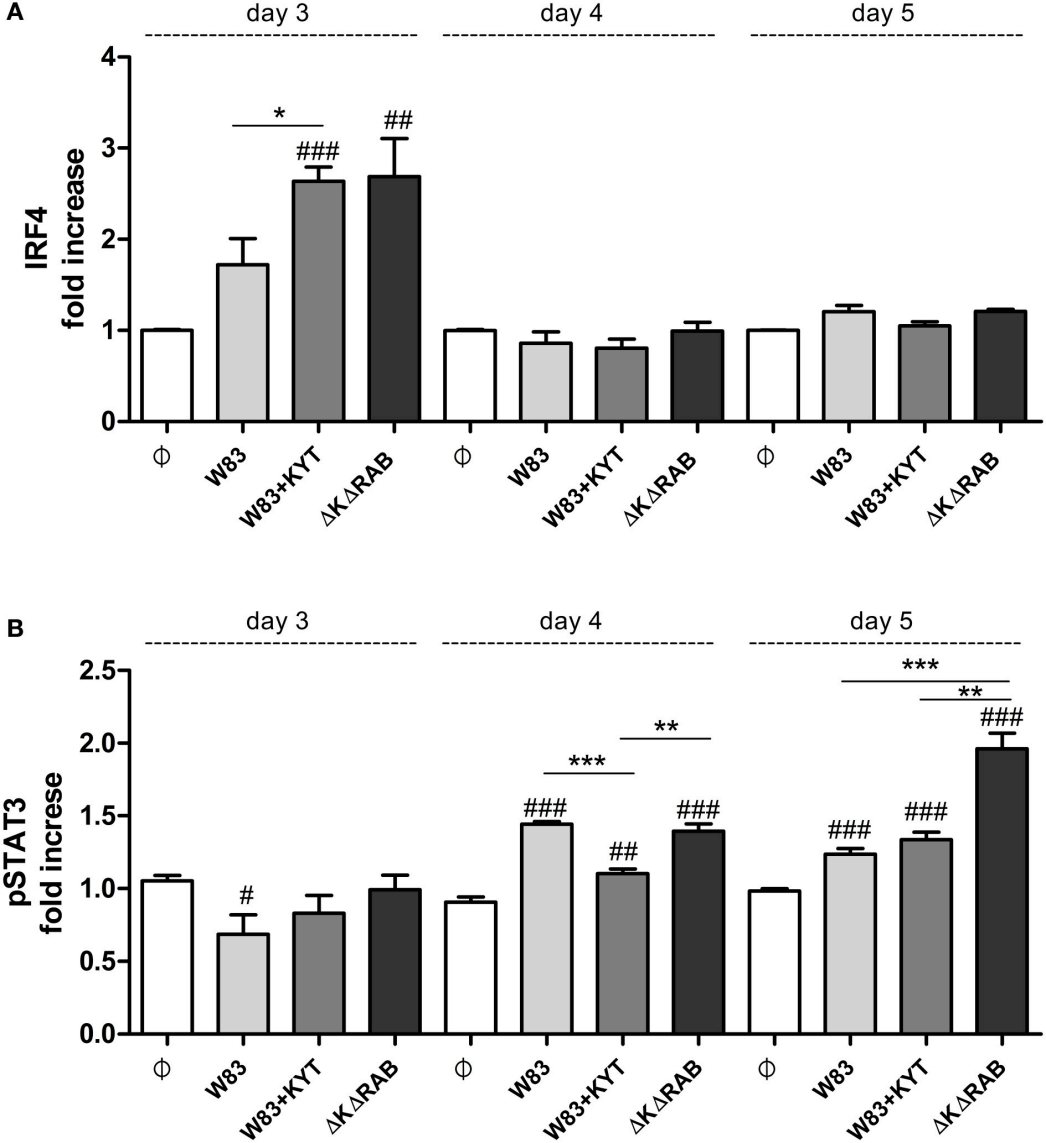
**Activation of STAT3 and IRF4 is dependent on gingipain activity**. At the end of each period, T cells were stimulated with PMA and ionomycin for 4 h in the presence of GolgiStop. Cells were stained for intracellular **(A)** IRF4 and **(B)** pSTAT3 (pY705), and IRF4, and analyzed by flow cytometry. Data show fold increase of the transcription factor positive population, relative to the appropriate control set at 1. Data are presented as a mean ± standard deviation of triplicate assays and were analyzed with a Student's *t*-test (^#^*P* < 0.05, ^##^*P* < 0.01, ^###^*P* < 0.001 vs. control, ^*^P < 0.05, ^**^P < 0.01, ^***^P < 0.001 to *P. gingivalis* treated cells).

To expand our studies, we evaluated some of the discrete events in the Th17 signaling. Firstly, we estimated the expression of positive regulators of the IL-17 in T cells, including receptors, adapter molecules, and transcription factors. The dynamic changes in the expression of particular molecules were observed between the first to the third day after T cells were exposed to moDCs infected with *P. gingivalis*. The most interesting finding was the initial upregulation of IRF-4 and BATF (day 1, 2), with subsequent induction of RORc, RORα and receptor for IL-1β (day 3; Figure 7). The highest expression was observed in infection with the *P. gingivalis* strain devoid of gingipains. Further analysis revealed activation of STAT3 and IRF-4, two transcription factors that play a major role in Th17 differentiation (Figures 8A,B). IRF-4 activity dominated in early post T cells co-culture with infected moDCs (day 3), whereas STAT-3 activity was delayed and its maximal effect was detected 2 days later (day 5). In both cases the activation level negatively correlated with gingipain activity. Collectively, we documented that the process of Th17 differentiation induced by *P. gingivalis* is negatively regulated by active gingipains.

### Inhibition of IL-6 signaling prevents gingipain-derived Th17 generation

**Figure 9:**
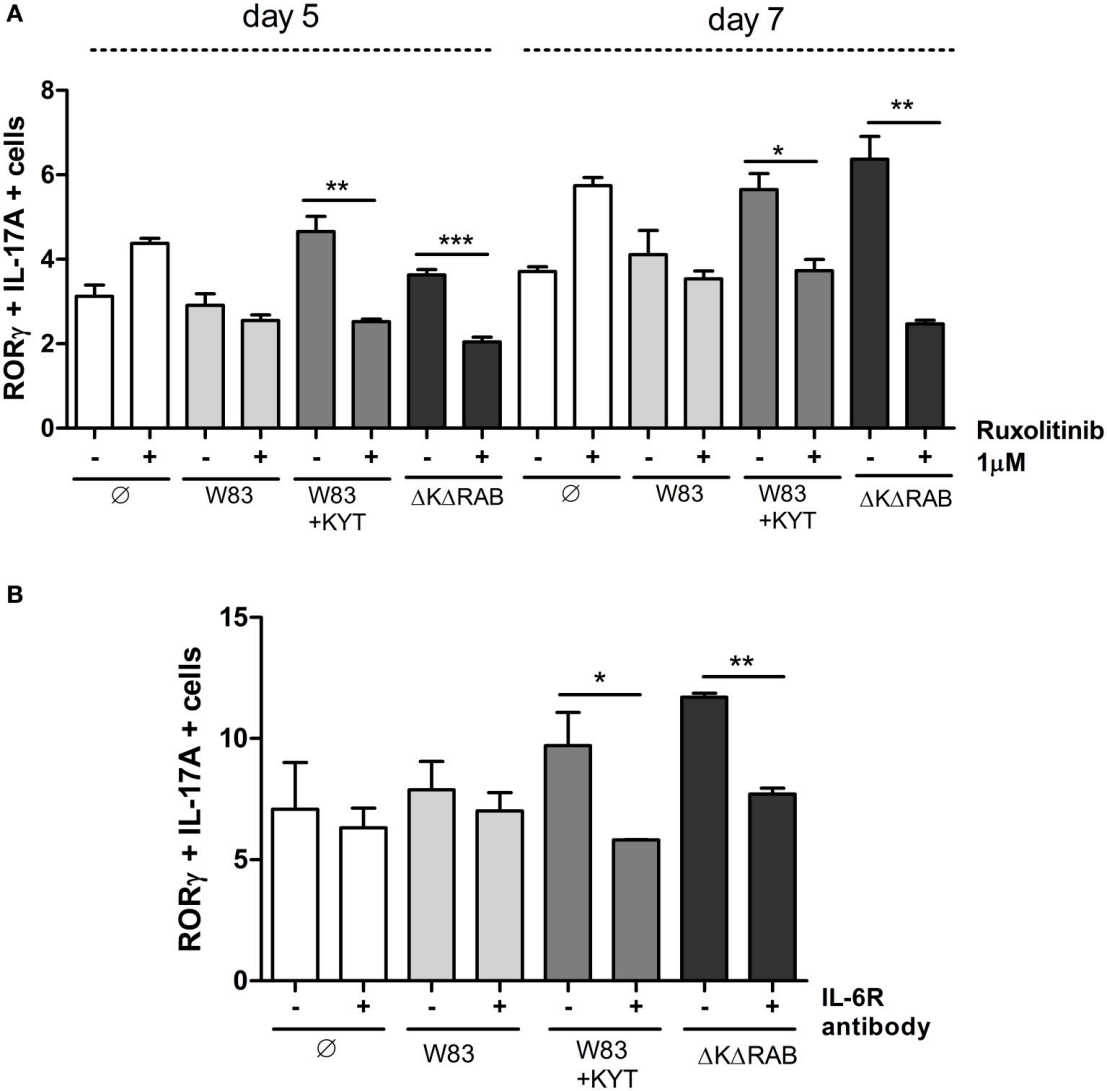
**Blocking the IL-6 signaling pathway results in reduction of the Th17 population stimulated by gingipains**. Monocyte-derived dendritic cells were untreated or exposed to *P. gingivalis* W83 in the presence/absence of specific protease inhibitors (KYT-1 and KYT-36, each at a concentration of 1 μM) for 6 h. Cultures were treated with the inhibitor of JAK1/JAK2 (Ruxolitinib [1 μM]) **(A)** or antibodies against the IL-6 receptor [10 μg/ml]) **(B)** for 2 h then naive CD4^+^ cells were added and cells were co-cultured for 5 or 7 days. At the end of each period, T cells were stimulated with PMA and ionomycin for 4 h in the presence of GolgiStop. For the evaluation of the Th17 population, cells were stained for IL-17A and RORγt, and analyzed by flow cytometry. Data show transcription factor and cytokine positive cells (RORγt^+^ IL-17A^+^) and are presented as a mean ± standard deviation of assays performed using three independent donors. Data were analyzed with a Student's *t*-test (^*^P < 0.05, ^**^P < 0.01, ^***^P < 0.001 to *P. gingivalis* treated cells).

IL-6 is one of the major and crucial cytokines responsible for generation of Th17 cells. IL-6 expression leads to cell activation via JAK kinases, and IL-6 mediated JAK2 phosphorylation activates STAT3, which stabilizes the transcription factor RORγt and promotes IL-17 expression. Therefore, we investigated the impact of IL-6 function on Th17 differentiation by selective blocking of the IL-6 signaling pathway. Ruxolitinib, an inhibitor of JAK kinases, was added to moDCs infected with *P. gingivalis* just before co-culturing with CD4+ T cells. Analysis for the Th17 phenotype revealed the ablation of their differentiation from naïve T lymphocytes, but only in the case of cells cultured with moDCs infected with the gingipain-null mutant or in the presence of KYTs (Figure 9A). The role of IL-6 signaling in gingipain-dependent differentiation of CD4+ T-cells was confirmed using antibody blocking of the IL-6 receptor that prevents formation of the IL-6/IL-6R complex (Figure 9B).

Collectively, our data revealed for the first time, that the process of Th17 differentiation induced by *P. gingivalis* is gingipain-dependent and is mediated via IL-6 expression. Moreover, we documented the role of enzymatically active gingipains from *P. gingivalis* in the negative regulation of the phenotype switch of CD4+ T helper effector cells and Th17 differentiation. Taken together, the inhibition of IL-6 signaling rather than gingipain activity might be a more appropriate target of treatment of chronic periodontitis since it leads to restoration of the low level of Th17 and regulation of its biological activity.

## Discussion

Th17 lymphocytes play an important role in the regulation of the immune response and bone resorption in a number of chronic diseases, such as rheumatoid arthritis and osteoarthritis (Wang et al., 2013). Recently, Th17 differentiation was also identified in periodontitis, the most prevalent form of bone pathology in humans afflicting up to 40% of the population in developed countries (Eke et al., 2012). Several clinical studies of chronic periodontitis have shown the presence of Th17 cells in the inflamed gingiva (Moutsopoulos et al., 2012). Interleukin-17 (IL-17) is a signature cytokine of Th17 cells. The IL-17 family of cytokines consists of several members such as IL-17A-F, which has emerged as key component of host defense responses and inflammatory diseases (Weaver et al., 2007). Although a substantial amount of data indicate that IL-17 plays a protective role in host defense against bacterial infections, it is also clear that excessive activation of the IL-17 pathway mediates connective tissue destruction and bone resorption (Lubberts, 2008). For instance, elevated levels of IL-17 were reported in human chronic periodontitis (Gaffen and Hajishengallis, 2008; Ohyama et al., 2009). Moreover, studies performed using a mouse model of periodontitis have revealed the significance of IL-17 for pathological bone loss (Eskan et al., 2012). It was found that upon aging, excessive expression of IL-17 led to spontaneous development of periodontitis, which was not observed in *IL-17R*–/– mice. The critical role of IL-17 in mediating bone loss was confirmed using a ligature model of periodontitis where local administration of mAbs to IL-17A or IL-17F inhibited bone destruction (Eskan et al., 2012).

Despite the undeniable role of IL-17 in the development of periodontitis, the molecular mechanism of Th17 differentiation underlying this phenomenon is not known. It is very likely that *P. gingivalis*, the major periodontal pathogen present in *circa* 73% of patients suffering from chronic periodontitis, is involved in Th17-dependent pathology. All characterized strains of *P. gingivalis*, as well as clinical isolates, produce gingipains (Ismail et al., 2015), which are major virulence factors capable of manipulation of a large number of defense and homeostatic mechanisms in infected tissue. Therefore, these cysteine proteases play a crucial role in progression of inflammation of the gingiva and tissue destruction (Guo et al., 2010; Ismail et al., 2015); however, their involvement in the regulation of Th17 differentiation has not been addressed.

The process of differentiation of naive CD4+ T cells into a particular subset of lymphocytes is regulated by cytokines expressed by activated antigen presenting cells (Luckheeram et al., 2012). Previous studies in humans have shown that elevated levels of some cytokines, such as IL-1β, IL-6, IL-21, and IL-23, are necessary for promoting Th17 differentiation and maintenance during the destructive phase of periodontitis (Takahashi et al., 2005; Moutsopoulos et al., 2012; Rao et al., 2015). Additionally, *in vitro* studies have reported the production of these Th17 supporting cytokines in cell cultures infected with *P. gingivalis* (Moutsopoulos et al., 2012). However, there are no data clearly showing the contribution of gingipains to this process. In the current study, we performed a complex analysis of the expression of Th17 relevant cytokines, both at the mRNA and protein levels, in cells exposed to *P. gingivalis*. To examine the role of gingipains in this process we stimulated professional (dendritic cells) and non-professional (gingival keratinocytes) antigen presenting cells with vital *P. gingivalis* in the presence or absence of specific inhibitors of gingipains (KYTs), as well as with an isogenic gingipain-null mutant entirely devoid of cysteine proteases (ΔKΔRAB). Surprisingly, we found that the inhibition of gingipain activity and deletion of all of the gingipain genes led to a significant increase of transcripts encoding IL-6, IL-21, and IL-23, which are crucial for Th17 polarization. The high levels of the IL-6 transcript correlated with the accumulation of the mature cytokines in conditioned medium but only in cultures infected with *P. gingivalis* depleted of gingipain activity by inhibitor treatment or genetic manipulation. Similar results were obtained with IL-8 and TNFα. These findings corroborate previous observations showing that heat-killed bacteria elicited production of a number of cytokines (Stathopoulou et al., 2009; Palm et al., 2013). Clearly, the proteolytically active gingipains strongly diminished the expression of cytokines at the transcriptional level. This down-regulatory effect of active gingipains on cytokine expression can be explained by their newly discovered ability to selectively proteolytically inactivate RIPK1, TAK1, and AKT kinases, which consequently impairs the immune response (Barth and Genco, 2016). Moreover, gingipains are known for their direct ability to degrade cytokines as IL-1β, IL-12, IL-6, and IL-8 (Banbula et al., 1999; Stathopoulou et al., 2009; Moutsopoulos et al., 2012), thus the lower level of the tested cytokines in the conditioned medium of cultures infected with untreated WT *P. gingivalis* can be explained by this mechanism. In fact, our data confirm that proteolytic activity of gingipains results in degradation of IL-6 (Banbula et al., 1999), which was clearly demonstrated using a reversible inhibitor of their activity (KYTs). Collectively, the data revealed that inactivation of gingipains led to strong upregulation of the Th17 relevant cytokines by at least a two pronged protease activity-dependent mechanism: inactivation of regulatory kinases and degradation of secreted cytokines. In this manner, gingipains strongly down-regulate the local inflammatory reaction. Conversely, gingipain inactivation strongly promotes activation of signaling pathways leading to expression of IL-6, IL-21, and IL-23 and thus the development of Th17 cells. Together, the results of our studies argue that the mechanism of Th17 differentiation in the gingiva should be more carefully studied. Although gingipains are not inhibited by any of human protease inhibitors, they are cysteine proteases and thus activity is dependent on a reducing environment. Therefore, it is conceivable that while diffusing away from the anoxic environment of periodontal pockets into oxygenated tissues they become enzymatically inactive due to oxidation of the thiol group of the catalytic cysteine residue, thus promoting the Th17 phenotype. The presence of gingipains in the deep in the gingival tissue (O-Brien-Simpson et al., 2003) lends support to this hypothesis. The balance of active and inactive gingipain may thus determine the composition of the local inflammatory milieu and have a strong influence on the scale of Th17 differentiation and progression of periodontitis.

In previous studies on the role of *P. gingivalis* in the differentiation of Th17, it was found that supernatants collected from *P. gingivalis*-infected myeloid APC were capable of expanding the Th17 population of CD4+ T cells, but not the Th1 population. Among all tested APCs, dendritic cells stimulated with *P. gingivalis* were the most effective in inducing Th17-differentiation (Moutsopoulos et al., 2012). To extend our findings, we also used monocyte-derived dendritic cells to explore the role of gingipains in Th17 differentiation. Moreover, in a deaparture from previous work (Moutsopoulos et al., 2012) we co-cultured infected moDC with a purified fraction of naive CD4+ T cells. In this system more closely resembling situation *in vivo*, we observed that bacterial infection significantly increased proliferation of lymphocytes. However, the rate of this process was substantially lower when the gingipain-null mutant (ΔKΔRAB) was used. In contrast to the previous observation (Moutsopoulos et al., 2012), we observed the induction of all tested CD4+ T cells subsets including Th1, Th2, and Th17. Notably, only the expansion of the Th17 cells population inversely correlated with the activity of gingipains, apparently related to the higher production of IL-6 and other related cytokines in co-cultures infected with the gingipain devoid bacteria. This is further augmented by the effect of inactive gingipains on several signaling components promoting IL-17/Th17 expression. Thus, it can be postulated that in contrast to inactive forms of gingipains, which are likely present in tissues distant from the periodontal pocket, enzymatically active gingipains in the anoxic tissue proximal to subgingival biofilm do not support maturation and differentiation of naive CD4+ lymphocytes into the Th17 population.

It is well-established that gingipains are primary virulence factors of *P. gingivalis* (Potempa et al., 2003) which determine disease progression in a murine model of alveolar bone loss (Pathirana et al., 2007; Wilensky et al., 2013). It is postulated that suppression of gingipain activity may reduce availability of nutrients and growth factors necessary for bacterial proliferation (Sroka et al., 2001; Imamura et al., 2003). In addition, it is generally accepted that inhibition of gingipains would render *P. gingivalis* susceptible to clearance by host defenses in the periodontal tissues (Olsen and Potempa, 2014). Therefore, these proteases seem to be a perfect target for development of novel therapeutics for periodontitis and associated systemic disorders, including cardiovascular disease, aspiration pneumonia, or pre-term birth low birth weight delivery (Olsen and Potempa, 2014). However, in light of our finding presented here it is clear that blocking the activity of gingipains with specific inhibitors may result in local up-regulation of immune responses and increased promotion of Th17 lymphocyte differentiation, which are associated with bone loss and chronic inflammatory responses. Importantly, our data evidently showed that IL-6 production was inversely correlated with the activity of gingipains. The presence of this cytokine is considered to be a marker of many chronic and autoimmune diseases such as Castleman disease, asthma, or multiple sclerosis (Yao et al., 2014). It is also well-defined that IL-6 plays a pivotal role in the bone pathology by its ability to promote the Th17 differentiation observed in rheumatoid arthritis and periodontitis (Yao et al., 2014). Together, these findings indicate caution in treatment of periodontitis by inhibition of gingipains, as this might have undesirable side effects.

Results of intensive studies on the biological activities of IL-6 and its pathological role imply that abrogation of IL-6 function and/or blockade of cytokine signaling pathways is a promising therapeutic strategy in inflammatory and autoimmune diseases (Yao et al., 2014). Thus, blocking of IL-6 signaling should be also considered as a potential strategy to treat periodontitis fueled by the inflammatory reactions propagated by periodontal pathogens. Indeed, Kobayashi et al. demonstrated recently that periodontal patients treated with Tocilizumab (TCZ), a recombinant humanized anti-human IL-6 receptor monoclonal antibody, displayed decreased inflammation in periodontal tissue (Kobayashi et al., 2015). This observation is in line with our finding here that antibodies against the IL-6 receptor significantly reduced the Th17 population. Moreover, IL-6 signaling activates the receptor-associated kinases JAK1, JAK2, and TYK2, which are responsible for the phosphorylation of STAT1 and STAT3. Activated STATs translocate to the nucleus and regulate the expression of numerous genes (Xiong et al., 2014). STAT3 is known as a crucial regulator of anti-apoptotic genes, and plays an important role in tumor growth, survival, and angiogenesis (Yu et al., 2009). In the case of the bone pathology, STAT3 is necessary for differentiation of Th17 cells, and regulates the expression of RORγt related to this population of lymphocytes. Furthermore, deficiency in STAT3 results in a decrease in RORγt expression and leads to increased levels of the T-bet and Foxp3 transcription factors which are characteristic for Th1 and Treg subpopulations, respectively (Yang et al., 2007).

Knowing that the JAK/STAT signaling pathway is involved in the regulation of CD4+ T cells we investigated the role of blocking JAK1/2 kinases on Th17 development induced by *P. gingivalis*. We applied Ruxolitinib, a newly discovered inhibitor of JAK1/JAK2 previously used in the treatment of myelofibrosis. Yajnanarayana et al. showed that Ruxolitinib is a potent inhibitor of Th1, Treg, and Th17 differentiation (Parampalli Yajnanarayana et al., 2015). In our model, we tested whether this inhibitor can abolish Th17 differentiation induced by an elevated level of IL-6 generated in response to *P. gingivalis*. Our data demonstrated that Ruxolitinib significantly reduced the percentage of RORγt and IL-17 positive cells, and thus IL-6 might be taken into consideration as a possible therapeutic agent for preventing bone loss during periodontitis.

In conclusion, this study revealed for the first time the complex role of cysteine proteases from *P. gingivalis* in IL-6 expression and Th17 differentiation. Active gingipains are modulators of immune responses significantly affecting the production of pro-inflammatory cytokines induced by other *P. gingivalis* virulence factors. On the other hand, the differentiation of naive CD4+ cells into the Th17 subpopulation is strongly enhanced by inactivation of gingipains, and promotes the development of the T lymphocyte phenotype typical of bone pathology. Taken together we propose that selective targeting of the IL-6 signaling pathway, rather than gingipain inhibition, might have a potential therapeutic value in treatment of periodontitis.

## Author contributions

IG and AW—the acquisition of data, drafting the work, approves the final version of manuscript, and agrees to be accountable for all aspects of the work. BP—the interpretation of data, drafting the work, approves the final version of manuscript, and agrees to be accountable for all aspects of the work. OB—the acquisition of data. ML and RL—the interpretation of data, revising the work, approves the final version of manuscript, and agrees to be accountable for all aspects of the work. JP—the analysis and interpretation of data, drafting the work, approves the final version of manuscript and agrees to be accountable for all aspects of the work. JK—the acquisition, analysis, and interpretation of data, drafting the work, approves the final version of manuscript and agrees to be accountable for all aspects of the work.

## Funding

This work was supported by National Science Center, Poland Grants 2011/03/B/NZ6/00053 (to JK), 2016/21/N/NZ6/01133 (to IG), and 2012/04/A/NZ1/00051 (to JP), who is also partially supported by Polish Ministry of Science and Higher Education 2975/7.PR/13/2014/2 (TRIGGER); JP and RL acknowledge support from NIDCR, grants R21DE023207 (to JP), and DE011111 and DE017921 (to RL). The Faculty of Biochemistry, Biophysics, and Biotechnology of the Jagiellonian University is a part of the Leading National Research Center programme supported by the Ministry of Science and Higher Education in Poland (KNOW, 35p/10/2016).

### Conflict of interest statement

The authors declare that the research was conducted in the absence of any commercial or financial relationships that could be construed as a potential conflict of interest.

## Supplementary material

Supplementary Figure 1 **Gingipain activity differentially determines the mRNA expression for Th17 supporting cytokines in TIGK cells**. Twenty four hours later, cells were lysed with TRIzol, RNA was isolated and reverse transcriptase PCR was performed. Relative expression of cytokine genes *IL-6, IL-21, IL-23* to the reference house-keeping gene *EF2* was measured by using Real-Time PCR. Data represents fold increase in expression compared to control levels, which were arbitrarily set at 1. Data are presented as mean ± standard deviation of assays performed three times, and were analyzed with a Student's *t*-test (#*P* < 0.05, ##*P* < 0.01 vs. control, ^*^*P* < 0.05, ^**^*P* < 0.01 to *P. gingivalis* treated cells).
