# Supplementary figures and images for: Inactive Gingipains from P. gingivalis Selectively Skews T Cells toward a Th17 Phenotype in an IL-6 Dependent Manner
Source: Front Cell Infect Microbiol. 2017 Apr 27;7:140. doi: 10.3389/fcimb.2017.00140 (PMC5406403; doi:10.3389/fcimb.2017.00140)

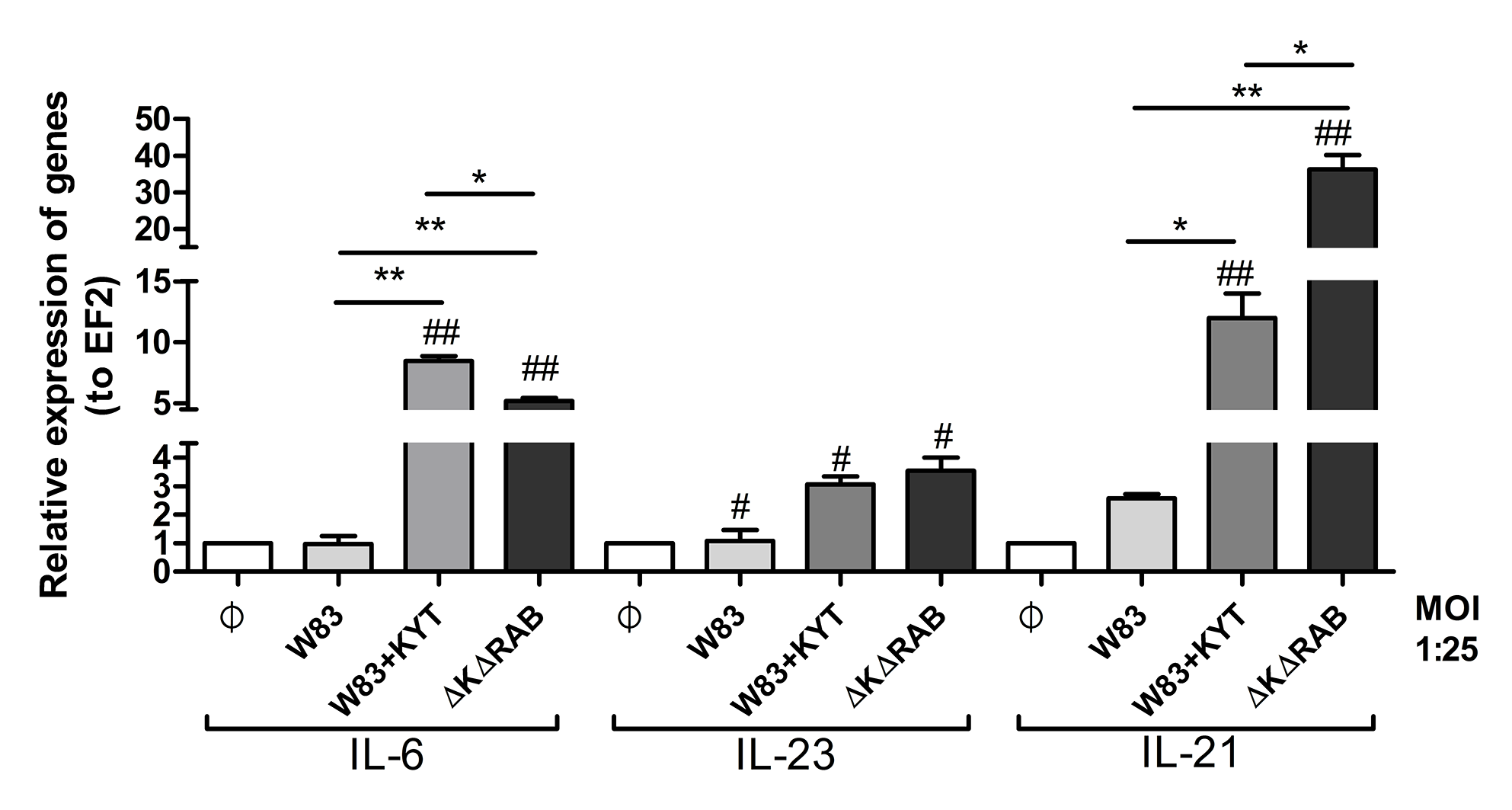

Supplement: Supplementary Figure 1 — Gingipain activity differentially determines the mRNA expression for Th17 supporting cytokines in TIGK cells. Twenty four hours later, cells were lysed with TRIzol, RNA was isolated and reverse transcriptase PCR was performed. Relative expression of cytokine genes IL-6, IL-21, IL-23 to the reference house-keeping gene EF2 was measured by using Real-Time PCR. Data represents fold increase in expression compared to control levels, which were arbitrarily set at 1. Data are presented as mean ± standard deviation of assays performed three times, and were analyzed with a Student's t-test (#P < 0.05, ##P < 0.01 vs. control, *P < 0.05, **P < 0.01 to P. gingivalis treated cells). [file Image1.TIF]
